# Encrypted Web traffic dataset: Event logs and packet traces

**DOI:** 10.1016/j.dib.2022.108188

**Published:** 2022-04-21

**Authors:** Stanislav Špaček, Petr Velan, Pavel Čeleda, Daniel Tovarňák

**Affiliations:** a Faculty of Informatics, Masaryk University, Brno, Czech Republic; b Institute of Computer Science, Masaryk University, Brno, Czech Republic

**Keywords:** HTTPS dataset, TLS 1.2 encryption, Host-based data collection, Network data collection, Encrypted traffic analysis, Event-flow correlation

## Abstract

We present a dataset that captures seven days of monitoring data from eight servers hosting more than 800 sites across a large campus network. The dataset contains data from network monitoring and host-based monitoring. The first set of data are packet traces collected by a probe situated on the network link in front of the web servers. The traces contain encrypted HTTP over TLS 1.2 communication between clients and web servers. The second set of data is an event log captured directly on the web servers. The events are generated by the Internet Information Services (IIS) logging and include both the IIS default features and custom features, such as client port and transferred data volume. Anonymization of all features in the dataset has been carefully carried out to prevent private information leakage while preserving the information value of the dataset. The dataset is suitable mainly for training machine learning techniques for anomaly detection and the identification of relationships between network traffic and events on web servers. We also add tools, settings, and a guide to convert the packet traces to IP flows that are often preferred for network traffic analysis.

## Specifications Table

**Table:** 

Subject	Computer Networks and Communications
Specific subject area	Network and host-based monitoring of encrypted web traffic in a campus network.
Type of data	Network traffic captured as packet traces divided into multiple PCAP files.
	Events logged by web servers structured as a newline-delimited JSON (JSON Lines) file.
	Python scripts used for anonymization of the data capture.
	Guide and settings for exporting IP flows from the packet traces.
How data were acquired	The data were acquired during normal operation in the campus network of Masaryk University, Brno, Czech Republic. Collection of network traffic and web server logs took place for seven days, from the 30th of July to the 6th of August 2021.
Data format	Raw
Description of data collection	Network traffic was captured by a single probe situated to capture all communication between clients outside and web servers inside the university network. The traffic was captured as packet traces and then split into PCAP files; one for each day of the monitoring. Events were collected from the Microsoft Internet Information Services 8.5 web server logs using the Syslog protocol. Then they were transformed into a unified JSON format and stored as a JSON Lines file.
Data source location	Masaryk University
	Brno
	Czech Republic
Data accessibility	Repository name: Science Data Bank
	Data identification number (DOI): 10.57760/sciencedb.01676
Direct link to the dataset:	https://www.scidb.cn/en/detail?dataSetId=ec72229d46624e4e8b8528dd0485f5b4

## Value of the Data

• Research and development of security and analytical methods in the field of cybersecurity depend on the availability of current and real operational data. However, obtaining real data is an issue because few researchers publish it due to fears of sensitive data leakage and exposing private information about users or the entire organization. To evade this problem entirely, researchers often use either old datasets, which no longer accurately represent the current situation, or non-public datasets, making replicating their results difficult or impossible. We publish our carefully anonymized dataset of current web traffic from a real campus network to provide the research community with up-to-date public data.
• The dataset is intended for researchers in the field of cybersecurity, performance measurement, and encrypted traffic analysis in need of comprehensive primary data representing current web traffic.
• Use-cases of the dataset include but are not limited to analysis of encrypted network traffic, behavioral analysis of web servers and their clients, identifying relations between events logged on web servers and network traffic, and learning and evaluating machine-learning algorithms for anomaly detection.
• Web traffic in the dataset is captured simultaneously from two angles – the network and host-based monitoring – and all devices were time-synchronized with millisecond precision. Consequently, it is possible to examine relationships between network traffic and events captured on web servers.
• The dataset has been carefully anonymized and stripped of any data that could lead to the identification of a specific person or the disclosure of the content of the communication. However, we paid special attention when considering what features to encode or cut from the dataset to prevent excessive loss of information value. We describe the anonymization process in detail in this article. Aside from anonymization, no filtering has been carried out, and the dataset accurately represents the encrypted HTTP over TLS 1.2 web traffic.
• IP flows are often preferred over the packet traces to represent network traffic for their efficiency in both research and production environments. The raw network traffic can be converted to IP flows in various ways. The accuracy of the original traffic representation depends directly on the flow export method. To expand and simplify the use of our dataset, we provide the packet traces as the most accurate network traffic representation. We also describe the process and provide the tools for converting the packet traces to IP flows. Consequently, everyone can easily customize the export method to fit their needs.

## 1 Data Description

The data was collected in a university network, where eight web servers provide access to more than 800 unique websites. Most of the traffic is comprised of standard user interactions with the sites. The clients access resources on web servers, and the servers respond to their requests. All these interactions with clients, such as processing, rejection, and redirection of requests, are recorded as events and stored on the web servers. In addition, a network traffic monitoring probe was placed on the line in front of the web servers. The probe captured all network data transmitted between clients and servers. This host-based and network monitoring data, which express two orthogonal views of web traffic, we captured over a period of seven days from the 30th of July to the 6th of August 2021. We filtered and anonymized the capture, and the resulting data is the content of this dataset.

In contrast to other available datasets, this dataset provides both the network data and events generated on web servers. It thus provides a more comprehensive view of the monitored web services. Both the network traffic probe and the web servers were time-synchronized, so it is possible to correlate network and host-based data based on the time of occurrence down to the level of packets (possibly aggregated into IP flows) and individual events. We see two main benefits of the event-flow correlation. The first benefit is the mutual consistency check, where attackers can modify events on a compromised server but cannot easily hide their network traffic. The second benefit is an enrichment of encrypted traffic monitoring using information from correlated events not affected by encryption. The benefits of event-flow correlation are supported by our previous research focused on the DNS protocol [1], [2].

### 1.1 Data format

We describe the structure and the features of the data included in the dataset. The dataset has two distinct parts – the host-based and the network monitoring data. The host-based data is stored in a single file. Due to its size, the network data is divided into separate files where each one represents one day of the data capture.

**Fig. 1:**
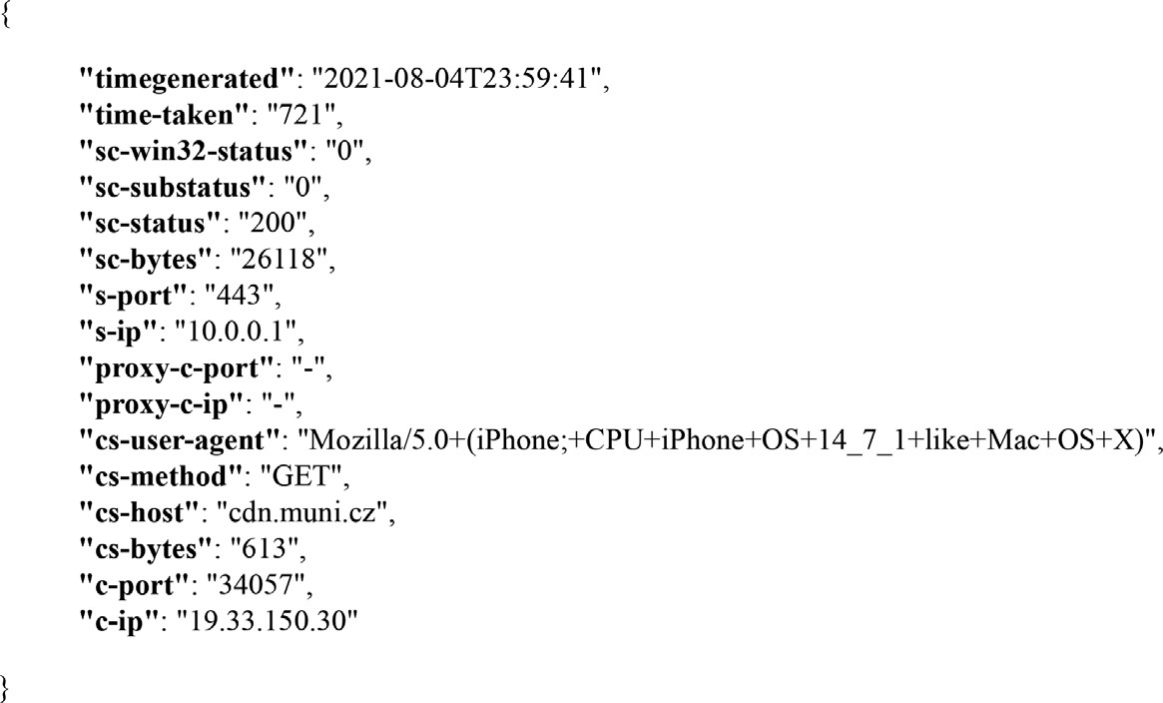
An example of an event from the host-based data.

The host-based data are represented by events gathered from web service logs maintained on each monitored server. They are provided in the JSON format and collected in a single JSON lines file [3]. All the events uphold the same format and comprise of the same feature set; they only differ in values contained therein. The format is shown on an example event in Fig. 1. The event features correspond to the W3C logging standard used by the IIS, and we refer to the documentation for their detailed description [4]. We only detail the time of occurrence feature and custom features that were added beyond the scope of the default IIS logging configuration using the enhanced logging [5].

The time of occurrence is logged in the UTC+2 time zone for all events in the dataset. Furthermore, time logging has two specific properties in IIS that deserve more detailed comments. The first property is that the timestamp stored in the *timegenerated* feature indicates the point in time when the server had resolved the request that generated the event. The request arrival timestamp can be calculated by subtracting the *time-taken* feature from the *timegenerated* feature. The *time-taken* feature contains the time interval the server spent processing the request (in milliseconds). The second property is the low precision of time measurement of the IIS web service. Even in its latest version 8.5, the IIS does not allow time tracking with better precision than in seconds. This is very inaccurate for a high-traffic environment. We found it almost impossible to increase the precision because it would require a significant extension or change to the entire web service logging system.

The first custom feature added through the enhanced logging is the *c-port*, which indicates the client port from which web communication was initiated. In default IIS logging, this feature is not monitored on web servers. However, from the point of view of network monitoring, it is one of the basic features distinguishing different IP flows. Current browsers usually open multiple parallel connections between a server and a client within a single session to speed up the loading of web pages. Without knowing the client port, it is impossible to distinguish which events were triggered by which connection within one specific session. Therefore we consider the client port to be a critical link between network data and host-based data, and it proved to be a vital feature impacting event-flow correlation [2], [6].

The other two custom features are *proxy-c-ip* and *proxy-c-port*, which we started monitoring due to the presence of reverse-proxy sites on the university network. If a website is behind a reverse proxy, an event on a web server is logged so that the reverse proxy acts as the client. This leads to inconsistencies between network and host-based data, where network traffic captures the original client, and the event captures the proxy. IIS 8.5 allows eliminating this inconsistency by passing the data about the original client from proxy to the web server, which is done via the *proxy-c-ip* and *proxy-c-port* features. When these features contain a value for a logged event, then the request passed through a reverse proxy, and the *proxy-c-ip* and *proxy-c-port* must be used when correlating with network traffic instead of the *c-ip* and *c-port*.

The last two custom features are *cs-bytes* and *sc-bytes*. The *cs-bytes* feature expresses the number of bytes transferred from the client to the server, *sc-bytes* analogously in the opposite direction. We consider these features important because they express how much data has been transferred over the network to meet a specific request. This value should correspond to the amount of data captured by the probe while considering the overhead for network transmission. Therefore, the volume of transferred data is another point of contact for the correlation of network and host-based data.

**Fig. 2:**
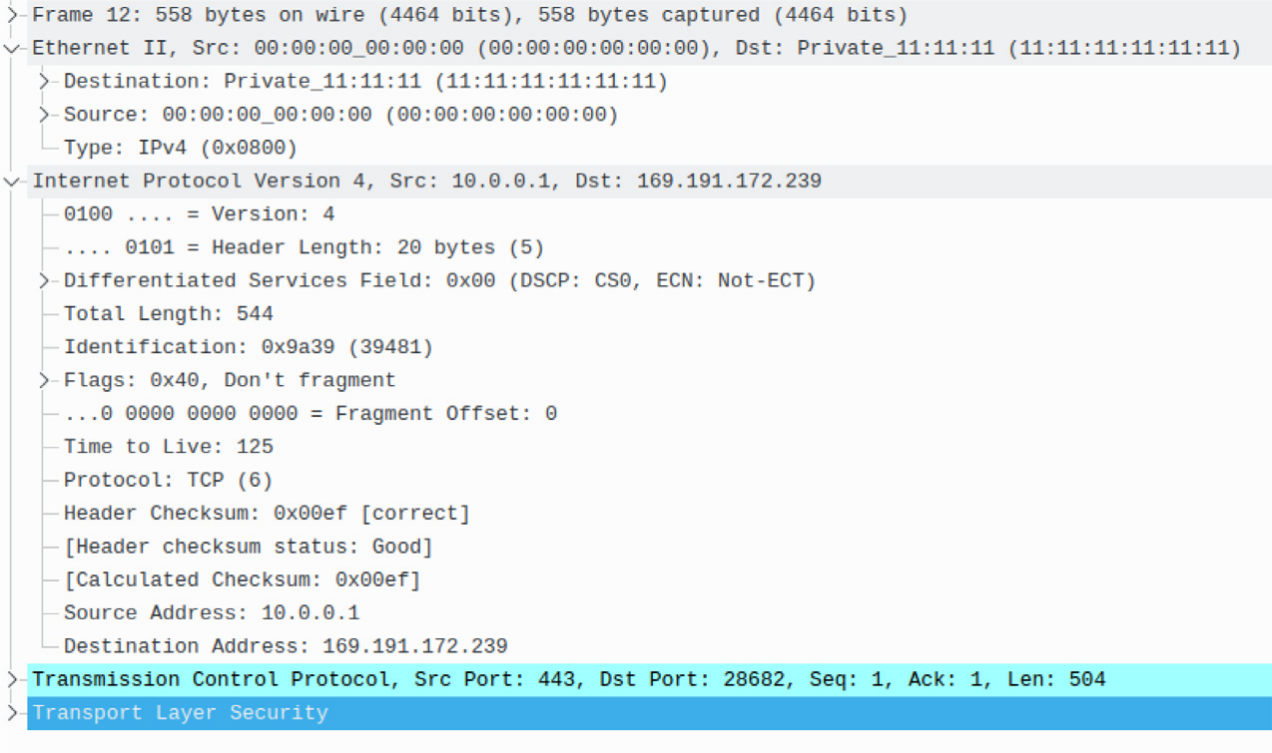
An example of a packet from the network traffic data.

Network data capture is stored in the libpcap format version 2.4 used by the tcpdump packet capture tool. Packet timestamps are captured and stored with microsecond precision [7]. Each packet has three layers (see Fig. 2): Ethernet layer, IPv4 layer, and TCP layer. The payload of the TCP protocol is always TLS protocol containing the HTTP traffic. The encrypted payload was not trimmed. The anonymization was performed only on the Ethernet and IP layers. The encrypted part of the traffic was kept in its original state.

### 1.2 Data repository structure

The repository contains the following folders:

• **Anonymization** – the anonymization folder contains the scripts and settings that were used to anonymize the data capture.
• **Dataset** – the dataset folder contains the host-based and network parts of the dataset in separate files.
• **Pcap2flow** – the pcap2flow folder contains the tools, settings, and the guide to convert the packet traces into IP flows.

## 2 Methods

The methods we used to capture, process, and publish the initial data capture as a dataset were designed with two goals in mind. The first goal was to provide an up-to-date and real web traffic dataset with high information value and minimal changes to the raw data. The second goal was not to disclose any private information along with the data. The anonymization process was somewhat simplified by the already encrypted HTTPS content of the web network traffic. Nevertheless, the initial capture still contained a large amount of client-specific information that had to be appropriately anonymized.

Our methodology can be summarized in the following points:

• We provide the HTTPS data as captured, without filtering and cleaning. Anomalies have not been remedied so that the dataset might contain monitoring errors or abnormal HTTPS communication.
• We only remove data from the dataset where it is required by our anonymization process defined in Section 2.2.
• We describe all operations performed on the data in the text of this article. A detailed description of these operations is provided in the form of Python scripts along with the dataset.

### 2.1 Data collection

**Fig. 3:**
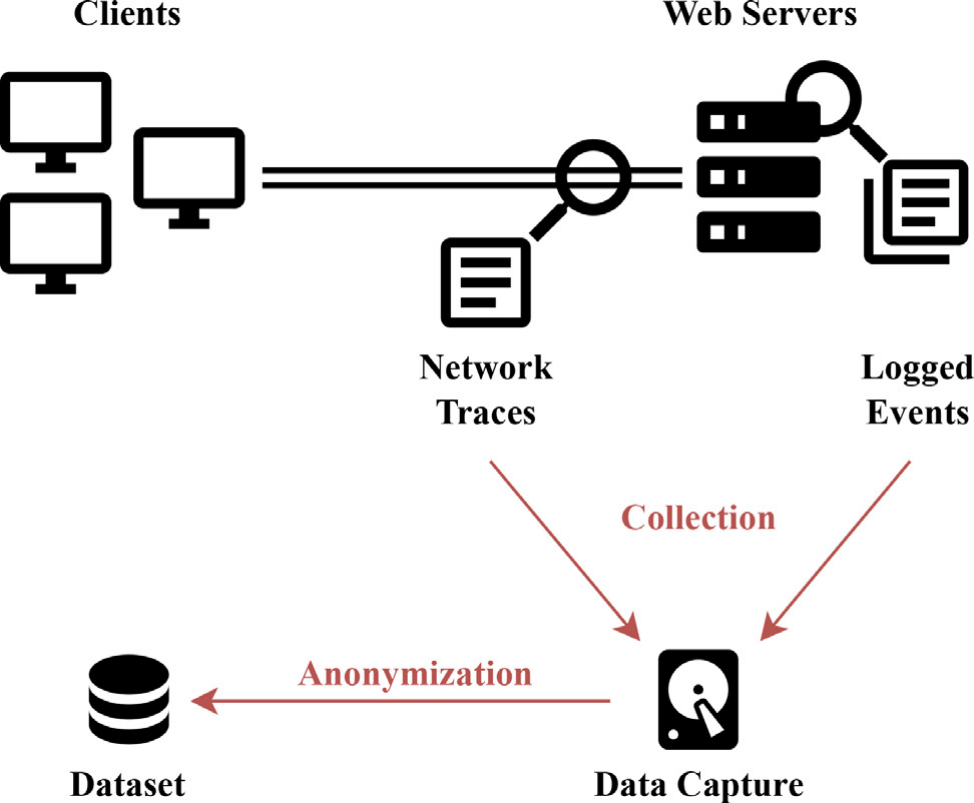
Data processing scheme – capture, collection, and anonymization.

Data collection took place on the campus network of Masaryk University, where we operate a monitoring infrastructure for anomaly detection in network traffic and server logs. We used only part of the whole infrastructure to monitor the web servers and their network traffic. The diagram of this part of the infrastructure is shown in Fig. 3. The image shows a network link between clients accessing the websites of Masaryk University and the servers where the websites are hosted. A probe monitoring network traffic is located on this network link, right at the edge of the campus network. The probe stores the network traffic locally as packet traces in PCAP files. Events are monitored and stored directly on individual web servers in their local logs. All the monitoring devices – the web servers and the probe – are time-synchronized by Network Time Protocol (NTP) with millisecond precision.

Network traffic capture was performed on a network probe equipped with an NFB-100G2 Field-Programmable Gate Array (FPGA) network card from Netcope Technologies. The card has two 100 GbE capable interfaces, but they are configured as 40 GbE interfaces in our setup. Both directions of traffic are received by this Network Interface Card (NIC), which allows us to capture both directions simultaneously. Timestamps for packets are provided by the NIC itself, which guarantees high precision. We have used a modified *libpcap* library with support for this card together with a standard *tcpdump* tool for the packet capture. Traffic was filtered based on known IP addresses of the web servers and on port 443, which is expected to contain TLS traffic. We rotated the capture file each hour to avoid filling up the small drive of the probe and to be able to transfer the files to another device for processing. After the packet capture was complete, we combined the partial files using the *mergecap* tool. After that, we used *reordercap* to ensure that all packets in the capture were ordered by their timestamps. Due to the large size of the final PCAP file, it was necessary to split it again before uploading it to the dataset repository. We decided to split it into individual days at midnight. The original file can be easily recovered using a *mergecap* command.

Web servers in our environment provide web services using the Internet Information Services (IIS) 8.5. Logging on the servers has been left in the default settings, except for adding custom features as described in Section 1.1. There is also a custom agent running on each web server. Its function is to collect all generated events at the end of each day and send them to the central log server. The central log server receives this data and immediately converts it to the JSON format. Finally, the events are stored and archived in a central data storage.

The result of the data collection phase is the data capture. It is a complete capture of network data and events limited to seven days from the 30th of July to the 6th of August 2021. It was created by collecting raw network data and events from the central repository into two files. The data capture contains all the information necessary to create our dataset. However, before transforming the data capture into the final dataset, anonymization was required.

### 2.2 Data anonymization

When creating the dataset from our data capture, we had to find a trade-off between the information value and the level of anonymity. Complete anonymization of the dataset would require breaking the links between flows and events triggered by a single client device. However, this would severely limit the use of the dataset; it would not be possible to investigate the correlation between IP flows and events. We have used separate anonymization processes for flows and events in the dataset. They are designed to remove the content of the communication from the dataset and remove or encode any identifiers that could reveal the identity of any user. However, they maintain the links between the data at the same time. In this chapter, we summarize both processes, but they can be examined in detail in the form of Python scripts in the repository folder *anonymization*.

The network traffic contains HTTP over TLS communication; therefore, the payload is not anonymized as it is already encrypted. The collected PCAP files were anonymized in two steps. First, VLAN information was dropped since it does not provide relevant data to the dataset. Second, the IP addresses and MAC addresses were replaced using the Python packet manipulation tool Scapy [8]. The source and destination MAC addresses in every packet were changed to 00:00:00:00:00:00 and 11:11:11:11:11:11, respectively. The IP addresses were handled separately for a client and a server and were substituted by their anonymized form in the dataset. Client IP addresses were anonymized using CryptoPAN [9]. Server addresses were replaced dynamically by addresses from the 10.0.0.0/24 range.

The event anonymization is more complicated because network traffic encryption does not affect them. Therefore, it is necessary to anonymize the identity of the client and the content of the communication. The events in the dataset were anonymized in four steps.

In the first step, we reduced the set of events only to those related to HTTPS traffic. The web servers accept and log HTTPS (server port 443) and a low volume of HTTP (server port 80) connections. HTTP connections are not the focus of this dataset, so events captured on a port other than 443 were dropped from the dataset.

In the second step, the client’s IP addresses were anonymized. Events caused by clients from the Masaryk University internal IP range (147.251.0.0/16) were dropped from the dataset. The list of university students and employees is public and the risk of at least partial data deanonymization is too high. For this reason, we decided not to include internal communication with web servers in this dataset. Client IP addresses outside the internal range were anonymized using the same CryptoPAN approach used for PCAP client IP anonymization to preserve event-flow relations.

In the third step, we focused on the event features that represent the content of the communication. In IIS logging, the content is represented by features *cs-uri-stem, cs-uri-query, cs-referer*, and *cs-username* [4]. The features contain specific queries, accessed resources, and possibly usernames. To mitigate the risk of deanonymization, these features were dropped from the dataset.

In the last step, we anonymized the servers’ IP addresses. The real IP addresses of the web servers are publicly known, so we used a different method for encoding them than for encoding client IP addresses. Otherwise, we would provide examples of address mapping before and after anonymization and thus provide means of breaking the CryptoPAN cipher. Instead of using CryptoPAN, server addresses were replaced dynamically from the 10.0.0.0/24 range. Mapping is shared with the process used in the PCAP file anonymization.

We considered anonymizing two more features in both the events and the IP flows – the Server Name Indication (SNI) and the User-Agent string. For both features, encoding the content can hide the value of the feature while maintaining the links between events and flows.

The SNI identifies which domain a client connects to when it accesses a web server that runs more than one domain. By encoding the SNI, we wanted to prevent identifying which specific sites are run by which specific server. However, the network traffic contains unencrypted SNI values even outside its reserved feature, e.g., in the server certificates. The server certificates are sent to the client as part of the TLS handshake. Modifying the certificate in the dataset would cause the integrity of the PCAP files to break, which in our opinion, would reduce the value of the dataset. By themselves, the domains in our capture cannot identify any specific user or any specific content that is accessed. Consequently, the SNI feature remained in our dataset.

The User-Agent string identifies a device, including, for example, the browser and operating system versions. After analyzing the content of the User-Agent strings in our capture, we decided not to proceed with its anonymization. The User-Agent string does not contain any background data by which it would be possible to identify a specific user. Due to the anonymization of other features in the dataset, it is impossible to connect the User-Agent string with a specific user except in the case of a unique string. We checked the layout of the different User-Agent strings in our dataset, and because we did not find any outliers, we decided to keep the User-Agent string present in the data.

### 2.3 IP flow export

Packet traces represented in the PCAP format encompass all the data transmitted over a monitored link and thus describe network traffic in every detail. However, monitoring the full volume of network traffic – the deep packet inspection – has high demands on the capacity and processing speed of analytical tools. In addition, with an ever-increasing frequency of encrypted traffic, the packet traces contain a large amount of data that has limited informational value without the means of decryption. Therefore, raw network data is usually aggregated into IP flows in research and production environments. The IP flows provide the benefit of decreasing the processing and storage requirements and easier data manipulation. However, the aggregation of individual packets into IP flows is not straightforward, as the flow feature set and exporter settings must first be defined. These settings influence the quality and final features of the IP flows generated from the original network traffic.

The flow feature set affects what network traffic information remains available in the IP flows. The basic IP flows, as defined in the RFC 5470, only inform who communicated and when and how much data was transferred [10]. However, there are many IP flow extensions that allow exporting specific data concerning the communication protocols above the IP layer, such as DNS, HTTP, and TLS. The optimal flow feature set for export is different for each research use case, so providing pre-exported IP flows instead of raw packet traces could limit the utility of our dataset.

The flow exporter settings also significantly impact the quality of the information in IP flows. The settings directly influence how packets are distributed between flow records. For example, in our research of event-flow correlation, it was shown that the success of the correlation was significantly affected by the exporter’s flow expiration policy [6]. We emphasize that optimal flow exporter settings depend on the specific research goal and thus should be considered on a case-by-case basis.

To extend the impact of our dataset, we decided to publish raw network data and resources on how to generate aggregated IP flow from the data. We describe the tools and processes to export IP flows from captured packet traces. We also provide the tools, settings, and a more in-depth guide on how to run the flow export in the dataset repository in the folder *pcap2flow*. We decided to provide the tools in a Docker container, which allows us to prepare a convenient way for the flow conversion replication [11]. However, the PCAP to flow conversion can also be performed using other export tools and in any other custom environment. The two pieces of software used in the container to transform a PCAP file to flow records are the *ipfixprobe* and the *IPFIXcol2* [12], [13].

The container specification can be examined in the Dockerfile and helper files. The conversion is straightforward; it starts the collector, runs the flow exporter on the provided PCAP file, and stops the collector after processing the PCAP. The flow exporter, represented by the *ipfixprobe*, processes the PCAP file, creates flow records, and sends them to the *IPFIXcol2* collector. The configuration for *ipfixprobe* is provided in the convert.sh script. Additional exporter plugins can be enabled to provide better insight into processed traffic by generating more information about the traffic. The collector, *IPFIXcol2*, formats and saves the flow records to a file using a JSON lines file format. However, the *IPFIXcol2* has multiple different output options that can be used as well. The collector is configured using the startup-json.xml configuration file, and the output format of the IP flow records can be changed there. Furthermore, additional IPFIX elements from *ipfixprobe* may be added to flows by specifying them in the cesnet.xml file.

## Ethics Statement

As a Cyber Security Incident Response Team of Masaryk University (CSIRT-MU), we are responsible for monitoring the campus network. When operating and deploying new monitoring tools and collecting data, we always consider their impact on user privacy. While preparing our dataset, we have made every effort to ensure that the data cannot be misused to identify specific users or devices.

We collected only the data that can be captured by network and host-based monitoring of encrypted traffic. The captured data did not contain any information about specific users, but they did contain information identifying client devices. We do not know the identity of the participants, and we did not use any side channels that would disclose more information about them.

After collecting the data, we manually checked them for private information and identified data features for anonymization. We developed a process to anonymize any client-specific data we identified in our capture. The approach to data anonymization was discussed and approved by the open science experts at our university. When the anonymization process finished, the original data capture was destroyed so that it currently only exists in its anonymized form. With the assumption that our anonymization process is not reversible with reasonable time and effort investment, the data in our dataset are anonymous.

## CRediT authorship contribution statement

**Stanislav Špaček:** Conceptualization, Methodology, Software, Validation, Investigation, Data curation, Writing – original draft. **Petr Velan:** Methodology, Software, Investigation, Data curation, Writing – original draft. **Pavel Čeleda:** Conceptualization, Methodology, Writing – review & editing, Supervision, Project administration, Funding acquisition. **Daniel Tovarňák:** Methodology, Writing – review & editing.

## Declaration of Competing Interest

The authors declare that they have no known competing financial interests or personal relationships which have, or could be perceived to have, influenced the work reported in this article.

## Data Availability

Organizational Culture (Original data) (Mendeley Data).
